# Fragile X Mental Retardation Protein: To Be or Not to Be a Translational Enhancer

**DOI:** 10.3389/fmolb.2018.00113

**Published:** 2018-12-11

**Authors:** Thomas Maurin, Barbara Bardoni

**Affiliations:** ^1^Université Côte d'Azur, CNRS UMR7275, Institute of Molecular and Cellular Pharmacology, Valbonne, France; ^2^CNRS LIA “Neogenex”, Valbonne, France; ^3^Université Côte d'Azur, INSERM, CNRS UMR7275, Institute of Molecular and Cellular Pharmacology, Valbonne, France

**Keywords:** FMRP, Fragile X Syndrome, translational regulation, RNP complexes, G-quadruplex, Q-tRNA

The Fragile X Syndrome (FXS) is a neurodevelopmental disorder due to the silencing of Fragile X Mental Retardation 1 (FMR1; Maurin et al., 2014). Patients are affected by intellectual disability of variable severity and they can also display a wide array of behavioral alterations such as hyperactivity, attention deficit, anxiety, deficit of language and epilepsy. Interestingly, FXS patients show several Autism Spectrum Disorder (ASD)-like symptoms, including social dysfunction, hyperactivity, stereotypic movements, hand-flapping and hand-biting, speech delay, and a relative lack of expressive language ability. Overall, ~30% of patients with FXS meet the full diagnostic criteria for ASD (Harris et al., 2008), while over 90% of individuals with FXS display some ASD symptoms (Hernandez et al., 2009*)*. Indeed, FXS is considered a form of ASD (https://www.spectrumnews.org/news/fragile-x-syndromes-link-autism-explained/) and so are the murine models of this disease (Melancia and Trezza, 2018). The *FMR1* gene encodes the Fragile X Mental Retardation Protein, that harbors three canonical RNA-binding domains (KH1, KH2 and RGG-Box) in addition to a Nuclear Localization Signal (NLS) and a Nuclear Export Signal (NES; Bardoni et al., 1997). The protein is mainly localized in the cytoplasm where it is a component of Ribonucleoprotein complexes (RNPs) associated with polyribosomes (Maurin et al., 2014). In neurons, FMRP is associated to synaptic polyribosomes and is a component of RNA granules, the RNP complexes transporting mRNAs along dendrites and axons (Khayachi et al., 2018). The presence of FMRP in nucleus and in nucleolus has been documented (Okray et al., 2015), even if it is not clear if this participates to some neuronal functions other than nuclear export of mRNAs, as part of specific mRNPs shuttling between nucleus and cytoplasm (Bardoni et al., 2006; Maurin et al., 2014). Furthermore, FMRP is often considered a multifunctional protein not only due to its implication in various steps of RNA metabolism but also because of its interaction with cytoskeleton components (Abekhoukh and Bardoni, 2014; Maurin et al., 2014) and ion channels (Ferron, 2016; Castagnola et al., 2018). Overall, these findings suggest that FMRP may coordinate the various steps of RNA metabolism with other cellular functions. However, in a general manner, translational regulation is the function of FMRP that researcher in the field have mainly studied. In this regard, the recent manuscript “Fragile X mental retardation 1 gene enhances the translation of large autism-related proteins” by Greenblatt and Spradling (Greenblatt and Spradling, 2018) renews the dilemma concerning the function of FMRP (Maurin et al., 2014; Dahlhaus, 2018). Although the dogma “FMRP is a translational repressor” exists, an increasing amount of data published during the last 18 years indicate a more complex implication of this protein in translational control (see Maurin et al., 2014 for review). Indeed:

1. A subset of proteins – encoded by FMRP RNA targets – have been shown to escape FMRP-dependent translational repression in mouse and human brain, such as SAPAP, UNC13, KIAA1091, TP63, casein kinase 1 gamma 2, NAP-22 (Brown et al., 2001); Sod1 (Miyashiro et al., 2003; Bechara et al., 2009), Ascl1 (Fähling et al., 2009), Kv4.2 (Gross et al., 2011), NOS1 (Kwan et al., 2012), and Dgkk (Tabet et al., 2016);

2. The ability of FMRP to specifically bind the mRNA of Sod1 and its reduced association to polyribosomes in mouse Fmr1-KO cells was further confirmed *in vivo* (Bechara et al., 2009; Davidovic et al., 2011; Nolze et al., 2013) and *in silico* (Cirillo et al., 2013) after a first observation (Miyashiro et al., 2003). This led to the identification of a fragment in the Sod1 mRNA, named SoSLIP (Sod1 Stem Loops Interacting with FMRP) that is specifically bound by FMRP. Remarkably, SoSLIP is able to increase the translation level of a reporter protein, being an enhancer of translation *per se*, a property that is potentiated by the presence of FMRP (Bechara et al., 2009). This suggests that various components of the same ribonucleoproteic complex are important to define the FMRP molecular role;

3. Translational repression by FMRP in brain seems to be associated to development, as shown comparing the expression level of synaptic proteins at 17 PND and 45 PND in Fmr1-null mouse brain vs. wild type (Tang et al., 2015). Indeed, in cortex synaptosomal preparation the expression of those proteins is highly deregulated in mouse Fmr1-KO compared with controls only at 17PND. Furthermore, in some regions of the brain of adult FXS patients, translation rate is not different when compared with controls (Qin et al., 2013; Tomasi et al., 2018). Collectively, these findings suggest a spatio-temporal-dependent function of FMRP, as also shown for the translational regulation of Ascl1 that is enhanced in newborn mouse brain (Fähling et al., 2009) but repressed in mouse embryonic stem cells (Khalfallah et al., 2017). Another example of such a regulation is provided by the regionalized regulation of GRK4 expression by FMRP that is restricted to adult cerebellum (Maurin et al., 2015). We recently published a large list of mRNAs modulated by FMRP in specific brain regions of young mice (Maurin et al., 2018a). Not surprisingly, the overlap is limited with FMRP targets characterized in HEK cells (Ascano et al., 2012) while it is higher with targets obtained from CLIP using total mouse brain extracts (Darnell et al., 2011).

By using ribosome profiling, a recent study compared mRNAs associated to translating polyribosomes in mouse adult neural stem cells (aNSC) in the presence and in the absence of FMRP (Liu et al., 2018). In Figure 1, we display the results of our analysis of the overlap between the mRNAs that are found differentially translated in *Fmr1*-KO aNSC with FMRP targets previously found by HITS-CLIP (Darnell et al., 2011; Maurin et al., 2018a). On one side, we observed that 199 mRNAs displayed reduced translation in *Fmr1*-null cells (three of them are target of FMRP according to Darnell et al., 2011 and Maurin et al., 2018a, while seven were found only by Maurin et al., 2018a). On the other side, 200 mRNAs displayed increased translation in *Fmr1*-null cells [4 of them are targets of FMRP previously found by Darnell et al., 2011, 21 of them found by Maurin et al., 2018a and 26 were common to the two studies)] (Figure 1). Considering old and new data, we can conclude that FMRP is mainly a translational repressor, at least during mammalian development. Now, Greenblatt & Spradling show that in oocytes dFMR1 - the fly homolog of FMRP and of its two paralog FXR1P and FXR2P, members of the Fragile X Related Protein family (FXRP) (Drozd et al., 2018) - enhances rather than represses the translation of a subset of mRNAs. Thus, how is it possible to reconcile former data with the new findings? This is an important issue due to the critical role that translational control has in normal functioning of brain (Sossin and Costa-Mattioli, 2018). It is worth to underline that fly dFMR1 has to achieve by itself the function of all three mammalian FXS proteins even if it is not possible to exclude that each mammalian FXR protein has peculiar properties or tissue-specific functions, as it was shown for FXR1P muscle isoforms (Bechara et al., 2007; Davidovic et al., 2013; Herman et al., 2018). In addition, except FMRP, the respective role of the other members of the FXR family in translational regulation has not been studied in great details so far (Bardoni et al., 2006; Maurin et al., 2014; Drozd et al., 2018). Thus, for instance, it is possible that one of them (or all) behaves as a translational enhancer in mammalian ovary. Indeed, under certain stimulation conditions, FXR1P was already shown to behave as a translational enhancer in monocytic cell lines (Vasudevan and Steitz, 2007). Furthermore, in mammalian cells we cannot exclude that the absence of a FXR member can be compensated by the function of another protein belonging to the same family. This cannot happen in fly due to the presence of a single FXR gene. In conclusion, concerning the molecular function of FMRP, several aspects should be studied to focus on elements that could interfere with its function, in the future.

**Figure 1:**
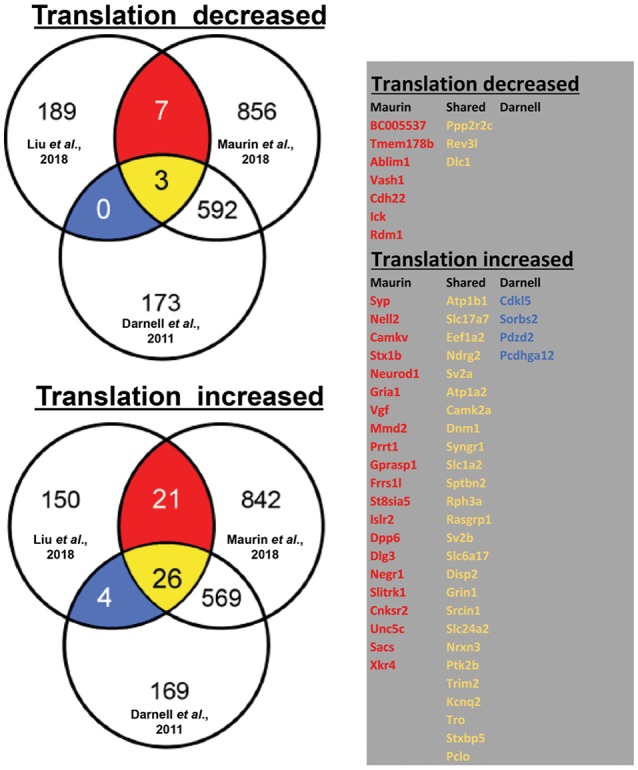
Translationally deregulated mRNA targets of FMRP. In the Venn diagrams (on the left), we present the overlap between mRNAs differentially associated to polyribosomes of WT and *Fmr1*-KO aNSC (Liu et al., 2018) with FMRP targets previously identified in total brain (Darnell et al., 2011) or in brain regions (Maurin et al., 2018b). To generate these data, we considered 10.716 transcripts that were found expressed in cells studied in all three works (considering as “expressed” those mRNA whose Log2 input counts were equal or greater than 0). Only 399/10.716 transcripts were observed as translationally deregulated in *Fmr1*- null aNSC (Liu et al., 2018) and then considered for the overlap with clipped mRNA (1236 from Maurin et al., 2018a and 682 from Darnell et al., 2011). The table (on the right) displays the identity of the translationally modulated mRNAs that have been already described as direct targets of FMRP (Darnell et al., 2011; Maurin et al., 2018b). We highlighted in red the target mRNAs found in the CLIP of Maurin et al., 2018a, in blue those found by Darnell et al., 2011, and in yellow those transcripts found in both studies.

Importantly, we and others showed that the basis of FMRP/RNA interaction is the key to understand its function (Darnell et al., 2001, 2011; Schaeffer et al., 2001; Bechara et al., 2009; Ascano et al., 2012; Suhl et al., 2014; Maurin et al., 2015, 2018a; Anderson et al., 2016) (see also 1). The questions are which RNAs are bound by FMRP in which tissues, at what time during development and how? It is not clear how to explain the function of FMRP and other RNA-binding proteins without knowing their RNA binding specificity. A key point to understand the molecular bases of the RNA/FMRP interaction is to establish whether this protein recognizes a structure (Figures 2A–C), and/or a sequence motif (Figures 2E,F). We have recently shown that short sequences that are common to target mRNAs of FMRP are recognized when in the context of a secondary RNA structure (Maurin et al., 2018a) as we had previously hypothesized studying the GRK4 RNA Interacting with FMRP (G4RIF) motif, bound by FMRP in the GRK4 mRNA (G protein-coupled Receptor Kinase 4; Maurin et al., 2015). We started from the structure-seq dataset in mouse embryonic stem cells (ES; Guo, 2016) and we assessed whether the motifs that we identified are engaged in Watson-Crick pairing in *vivo*. To do so, we computed an unpairing score for each base of each expressed transcript. We derived a score for each motif bound by FMRP and we compared the scores of motifs embedded in FMRP binding sites or present elsewhere in the transcript. Our analysis clearly shows that the CUGKA, GWRGA and UAY motifs present in regions bound by FMRP are more accessible to DMS modification *in vivo* than the unbound cognate motifs present in the same transcripts (Maurin et al., 2018a). This shows that FMRP prevalently recognizes motifs that are presented in single stranded regions or loop sequences of stem loop structures. Also, clusters of the WGGA motif identified by Ascano et al. (2012) were proposed to form RNA G-quadruplex forming structure in targets of FMRP (Suhl et al., 2014; Anderson et al., 2016). This finding is also supported by our results (Maurin et al., 2018a) showing an enrichment of the G-quadruplex structure in target mRNAs of FMRP. Collectively, these findings definitively suggest that FMRP recognizes and binds structural motifs. For instance, it was proposed that G-quadruplex forming structures can be stabilized by FMRP and block the polyribosomes scanning when they are located in the 5'UTR of a FMRP target mRNA (Melko and Bardoni, 2010), thus explaining the role of FMRP as repressor of translation. Furthermore, while FMRP recognizes structural motifs, sequences harbored by them might be critical for its translational action. Indeed, we and others (Anderson et al., 2016; Maurin et al., 2018a) have shown that FMRP binding sites located in mRNA coding regions are enriched for the GAC codon. This remained an unexplained feature of FMRP binding for a while but a recent report shines new light on this puzzling observation. The GAC codon is decoded by the m38C_tRNA _Asp, a highly modified tRNA harboring a GUC anticodon carrying a hyper modified Guanosine called Queuosine (Q). Q is only provided through the microbiota or food and therefore Q-tRNA may confer nutritional control of protein translation. In mammalian cells, Q deprivation stalls ribosomes at GAC codons and to a smaller extent at near-cognate codons (Tuorto et al., 2018). Collectively these findings lead to the speculation that the ability of FMRP to stall polyribosomes—one of the mechanisms proposed to explain the role of translational repressor of FMRP (Darnell et al., 2011; Richter and Coller, 2015)—could be related to m38C_tRNA _Asp metabolism. This could be achieved in several ways, for instance, FMRP could modulate a rate-limiting step of tRNA queuosinylation or, alternatively, may compete with this tRNA for its P site occupancy in the elongating ribosome (Chen et al., 2014). In this context, the percentage of GAC codons in mRNA coding regions could define the role of repressor of FMRP for them. Interestingly, with the exception of SoSLIP—enhancing the translation—other RNA motifs have been so far associated to the capacity of FMRP to repress translation (Darnell et al., 2001, 2005; Schaeffer et al., 2001; Ascano et al., 2012; Maurin et al., 2015, 2018a; Anderson et al., 2016). However, until now this latter function was the main studied, thus it is not surprising that the majority of motifs bound by FMRP are associated to its role as a translation repressor. We can only speculate that these molecular mechanisms are conserved in drosophila, as such molecular studies have not been published yet (Drozd et al., 2018; Greenblatt and Spradling, 2018).

**Figure 2:**
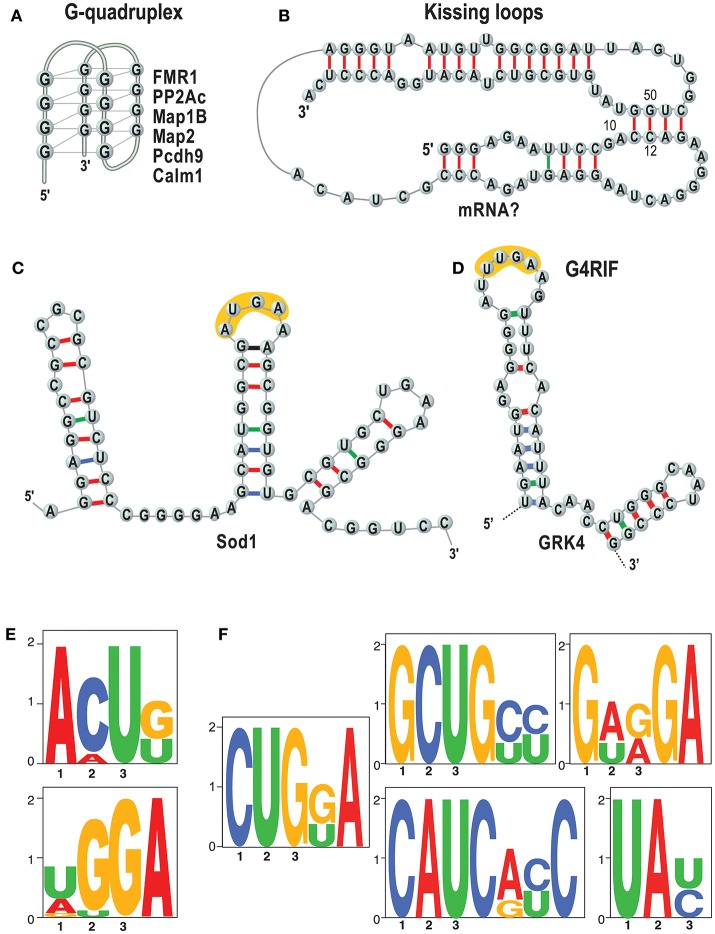
RNA motifs bound by FMRP. Main structures and sequences that are bound by FMRP are listed. **(A)** The G-quadruplex (Schaeffer et al., 2001) structure is represented and some targets of FMRP harboring a G-quadruplex are listed (Darnell et al., 2001; Schaeffer et al., 2001; Castets et al., 2005; Maurin et al., 2018a); **(B)** Kissing loop (Darnell et al., 2005). No natural mRNAs have been found so far harboring this structure. **(C)** SoSLIP was found in *Sod1* mRNA and it spans the AUG of this mRNAs, being also an IRES motif (Bechara et al., 2009). In yellow one of the short motifs identified by (Ascano et al., 2012) (see **E)** is indicated.; **(D)** G4RIF found in *GRK4* mRNA (Maurin et al., 2015). In yellow one of the short motifs identified by (Ascano et al., 2012) (see **E)** is indicated; **(E)** that were found by PAR-CLIP in HEK cells (Ascano et al., 2012). **(F)** Sequences that we have identified as enriched in RNA fragment bound by FMRP and resulting from an analysis of HIT-CLIP in various brain areas (Maurin et al., 2018a). The motif TAY was also indicated as main target of FMRP (Anderson et al., 2016) by comparing two previous CLIP assays (Darnell et al., 2011; Ascano et al., 2012) that were performed in total brain extracts and HEK cells, respectively.

The second key point that should be considered to explain the double role of FMRP as repressor and enhancer of translation is represented by the interactors of FMRP, which can have different expression patterns (Bardoni et al., 2006; Bonaccorso et al., 2015). These proteins can modify the RNA binding specificity of FMRP, as for instance FXR1P in brain (Bechara et al., 2007) and this may generate different mechanisms of action of this protein (as discussed at point 3). It is then possible that- considering the recent findings- the different function of dFMR1 in oocytes compared to brain results from the presence of tissue- specific FMRP interacting proteins. Furthermore, FMRP acts as a part of a ribonucleoproteic complex and its function should be considered in the context of RNPs having different roles in the cells (export from nucleus, transport at the synapse, transport between polyribosomes and stress granules or P-Bodies; Maurin et al., 2014) and, likely, regulated by different stimuli, such as mGlur5 stimulation in neurons (Khayachi et al., 2018). The identification of interacting proteins of FMRP that are able to modify its ability to recognize and bind mRNA can represent a clue to understand the multiple functions of FMRP, since in various subcellular compartments (e.g., nucleus, cytoplasm, synapse) different proteins are present.

Another critical point is represented by the observation that, in the absence of FMRP, a set of its mRNA targets was shown to be less transported at the synapse (Dictenberg et al., 2008), while it has been described that the dendritic transport of two of its target mRNAs is enhanced (Vicario et al., 2015; Maurin et al., 2018a). This suggests that, in the absence of FMRP, the increased or decreased level of translation could be due to an altered abundance of mRNA available to active translating ribosomes in soma and/or at the synapse due to an altered turnover of mRNA transported between nucleus and soma and (in neurons) between soma and synapses, rather than caused by (or in addition to) other molecular dysregulations. In our opinion, this element is also important to understand the results of Greenblatt & Spradling since fly oocytes are polarized cells in which mRNA distribution is tightly regulated, similar to neurons (Martin and Ephrussi, 2009).

Last but not least, the post-translational modification of FMRP can modify its function. For instance, sumoylation has been shown to have a strong impact on the ability of FMRP to interact with partners and generate complexes (Khayachi et al., 2018) as it involves a domain that is critical for protein/protein interaction (Bardoni et al., 1999; Adinolfi et al., 2003; Ramos et al., 2006).

In conclusion, it seems now the right time to renew the research on the mechanisms of action of FMRP. It is indeed remarkable that the study of Liu et al. (2018) (See Figure 1 legend) shows that the vast majority of FMRP mRNA targets (75%: 625/830 of Darnell's target dataset that are those associated to polyribosomes) *are not modulated in the abse*nce of FMRP, which in our opinion argues for a stronger implication of FMRP-containing mRNPs in storage/transport of mRNAs rather than in translational regulation. To date the role of FMRP was mainly studied in translational regulation probably due to its association to polyribosomes (Corbin et al., 1997; Khandjian et al., 2004) and consistent with the link existing between translation and mGluR-dependent Long Term Depression that has been reported to be exaggerated in *Fmr1*-KO (Huber et al., 2002). Given the particular role of FMRP at the synapse, it would be interesting to reproduce this analysis in the synaptic compartment, by taking advantage, for instance, of the single cell RNA technology to study the implication of FMRP in intracellular RNA trafficking or local translation (Pichon et al., 2018). It is easy to speculate that while motifs bound by FMRP and located in coding regions of mRNA are mainly associated to translational regulation, the motifs present in 3'UTR regions—more than 30% of FMRP target mRNA (Maurin et al., 2018a) harbor a motif in this region- are involved in processes of precise sub cellular location and/or maturation.

It is obvious that the function of this protein is intimately linked to the identification of a therapy for FXS, that, indeed, is still missing (Castagnola et al., 2017). In this context, it is remarkable to notice the new therapeutic target of FMRP, Phosphodiesterase 2A (PDE2A; Androschuk et al., 2018; Maurin et al., 2018b) has been identified by HITS-CLIP (Darnell et al., 2011; Maurin et al., 2018a) as well as, in the past the APP through the immunoprecipitation of FMRP RNP from mouse brain (Brown et al., 2001; Westmark et al., 2016). Furthermore, a Phase 3 clinical trial is ongoing using a combination of various anti-oxidants to treat FXS patients (NCT02942498), as suggested the fact that SOD1 is less expressed in mouse *Fmr1-*KO cells (Bechara et al., 2009) and, consequently, markers of oxidative stress have been described in brain of infant and adult *Fmr1*-null mice (El Bekay et al., 2007; de Diego-Otero et al., 2009; Davidovic et al., 2011).

## Author Contributions

The two authors listed have made a substantial, direct and intellectual contribution to the work, and approved it for publication.

### Conflict of Interest Statement

The authors declare that the research was conducted in the absence of any commercial or financial relationships that could be construed as a potential conflict of interest.

## References

[B1] Abekhoukh S. Bardoni B. (2014). CYFIP family proteins between autism and intellectual disability: links with Fragile X syndrome. Front. Cell. Neurosci. 8:81. 10.3389/fncel.2014.00081 24733999 PMC3973919

[B2] Adinolfi S. Ramos A. Martin S. R. Dal Piaz F. Pucci P. Bardoni B.. (2003). The N-terminus of the fragile X mental retardation protein contains a novel domain involved in dimerisation and RNA-binding. Biochemistry 42, 10437–10444. 10.1021/bi034909g 12950170

[B3] Anderson B. R. Chopra P. Suhl J. A. Warren S. T. Bassell G. J. (2016). Identification of consensus binding sites clarifies FMRP binding determinants. Nucleic Acids Res. 44, 6649–6659 10.1093/nar/gkw593 27378784 PMC5001617

[B4] Androschuk A. He R. X. Weber S. Rosenfelt C. Bolduc F. V. (2018). Stress odorant sensory response dysfunction in Drosophila Fragile X Syndrome mutants. Front. Mol. Neurosci. 11:242. 10.3389/fnmol.2018.00242 30135642 PMC6092503

[B5] Ascano M. Jr. Mukherjee N. Bandaru P. Miller J. B. Jeffrey D Nusbaum J. D.. (2012). FMRP targets distinct mRNA sequence elements to regulate protein expression. Nature 492, 382–386. 10.1038/nature11737 23235829 PMC3528815

[B6] Bardoni B. Davidovic L. Bensaid M. Khandjian E. W. (2006). The fragile X syndrome: exploring its molecular basis and seeking a treatment. Expert Rev. Mol. Med. 8, 1–16. 10.1017/S1462399406010751 16626504

[B7] Bardoni B. Schenck A. Mandel J. L. (1999). A novel RNA binding nuclear protein that interacts with the fragile X mental retardation (FMR1) protein. Hum. Mol. Genet. 8, 2557–2566. 10.1093/hmg/8.13.2557 10556305

[B8] Bardoni B. Sittler A. Shen Y. Mandel J. L. (1997). Analysis of domains affecting intracellular localization of the FMRP protein. Neurobiol. Dis. 4, 329–336. 9440121 10.1006/nbdi.1997.0142

[B9] Bechara E. Davidovic L. Melko M. Bensaid M. Tremblay S. Grosgeorge J.. (2007). Fragile X related protein 1 isoforms differentially modulate the affinity of fragile X mental retardation protein for G-quartet RNA structure. Nucleic Acids Res. 35, 299–306. 10.1093/nar/gkl1021 17170008 PMC1802556

[B10] Bechara E. G. Didiot M. C. Melko M. Davidovic L. Bensaid M. Martin P.. (2009). A novel function for fragile X mental retardation protein in translational activation. PLoS Biol. 7:e16. 10.1371/journal.pbio.1000016 19166269 PMC2628407

[B11] Bonaccorso C. M. Spatuzza M. Di Marco B. Gloria A. Barrancotto G. Cupo A. (2015). Fragile X mental retardation protein (FMRP) interacting proteins *et al*. exhibit different expression patterns during development. Int J Dev Neurosc. 42, 15–23. 10.1016/j.ijdevneu.2015.02.004 25681562

[B12] Brown V. Jin P. Ceman S. Darnell J. C. O'Donnell W. T. Tenebaum S. A.. (2001). Microarray identification of FMRP-associated brain mRNA and altered mRNA translational profiles in fragile X syndrome. Cell 107, 477–487. 10.1016/S0092-8674(01)00568-2 11719188

[B13] Castagnola S. Bardoni B. Maurin T. (2017). The search for an effective therapy to treat Fragile X Syndrome: dream or reality? Front. Synaptic Neurosci. 9:15 10.3389/fnsyn.2017.00015 29163124 PMC5681520

[B14] Castagnola S. Delhaye S. Folci A. Paquet A. Brau F. Duprat F.. (2018). New insights into the role of Ca_v_2 protein family in calcium flux deregulation in *Fmr1*-KO neurons. Front. Mol. Neurosci. 11:342. 10.3389/fnmol.2018.00342 30319351 PMC6170614

[B15] Castets M. Schaeffer C. Bechara E. Schenck A. Khandjian E. W. Luche S.. (2005). FMRP interferes with the Rac1 pathway and controls actin cytoskeleton dynamics in murine fibroblasts. Hum. Mol. Genet. 14, 835–844. 10.1093/hmg/ddi077 15703194

[B16] Chen E. Sharma M. R. Shi X. Agrawal R. K. Joseph S. (2014). Fragile X mental retardation protein regulates translation by binding directly to the ribosome. Mol. Cell 54, 407–417. 10.1016/j.molcel.2014.03.023 24746697 PMC4019695

[B17] Cirillo D. Agostini F. Klus P. Marchese D. Rodriguez S. Bolognesi B.. (2013). Neurodegenerative diseases: quantitative predictions of protein-RNA interactions. RNA 19, 129–140. 10.1261/rna.034777.112 23264567 PMC3543085

[B18] Corbin F. Bouillon M. Fortin A. Morin S. Rousseau F. Khandjian E. W. (1997). The fragile X mental retardation protein is associated with poly(A)+ mRNA in actively translating polyribosomes. Hum. Mol. Genet. 6, 1465–1472. 9285783 10.1093/hmg/6.9.1465

[B19] Dahlhaus R. (2018). Of men and mice: modeling the Fragile X syndrome. Front. Mol. Neurosci. 11:41. 10.3389/fnmol.2018.00041 29599705 PMC5862809

[B20] Darnell J. C. Fraser C. E. Mostovetsky O. Stefani G. Jones T. A. Eddy S. R.. (2005). Kissing complex RNAs mediate interaction between the Fragile-X mental retardation protein KH2 domain and brain polyribosomes. Genes Dev. 19, 903–918. 10.1101/gad.1276805 15805463 PMC1080130

[B21] Darnell J. C. Jensen K. B. Jin P. Brown V. Warren S. T. Darnell R. B. (2001). Fragile X mental retardation protein targets G quartet mRNAs important for neuronal function. Cell 107, 489–499. 10.1016/S0092-8674(01)00566-9 11719189

[B22] Darnell J. C. Van Driesche S. J. Zhang C. Hung K. Y. Mele A. Fraser C. E.. (2011). FMRP stalls ribosomal translocation on mRNAs linked to synaptic function and autism. Cell 146, 247–261. 10.1016/j.cell.2011.06.013 21784246 PMC3232425

[B23] Davidovic L. Durand N. Khalfallah O. Tabet R. Barbry P. Mari B.. (2013). A novel role for the RNA-binding protein FXR1P in myoblasts cell-cycle progression by modulating p21/Cdkn1a/Cip1/Waf1 mRNA stability. PLoS Genet. 9:e1003367. 10.1371/journal.pgen.1003367 23555284 PMC3605292

[B24] Davidovic L. Navratil V. Bonaccorso C. M. Catania M. V. Bardoni B. Dumas M. E. (2011). A metabolomic and systems biology perspective on the brain of the fragile X syndrome mouse model. Genome Res. 21, 2190–2202. 10.1101/gr.116764.110 21900387 PMC3227107

[B25] de Diego-Otero Y. Romero-Zerbo Y. El Bekay R. Decara J. Sanchez L. Rodriguez-de-Fonseca F. (2009). Alpha-tocopherol protect against oxidative stress in the fragile X knockout mouse:an experimental therapeutic approach for the Fmr1 deficiency. Neuropsycopharmacology 34, 1011–1026. 10.1038/npp.2008.152 18843266

[B26] Dictenberg J. B. Swanger S. A. Antar L. N. Singer R. H. Bassell G. J. (2008). A direct role for FMRP in activity-dependent dendritic mRNA transport links filopodial-spine morphogenesis to fragile X syndrome. Dev. Cell 14:926–939. 10.1016/j.devcel.2008.04.003 18539120 PMC2453222

[B27] Drozd M. Bardoni B. Capovilla M. (2018). Modeling fragile X syndrome in drosophila. Front. Mol. Neurosci. 11:124. 10.3389/fnmol.2018.00124 29713264 PMC5911982

[B28] El Bekay R. Romero-Zerbo Y. Decara J. Sanchez-Salido L. Del Arco-Herrera I. Rodriguez-de-Fonseca F.. (2007). Enhanced markers of oxidative stress, altered antioxidants and NADPH-oxidase activation in brains from Fragile X mental retardation 1-deficient mice, a pathological model for Fragile X syndrome. Eur. J. Neurosci. 26, 3169–3180. 10.1111/j.1460-9568.2007.05939.x 18005058

[B29] Fähling M. Mrowka R. Steege A. Kirschner K. M. Benko E. Forstera B.. (2009). Translational regulation of the human achaete-scute homologue-1 by fragile X mental retardation protein. J. Biol. Chem. 284, 4255–4266. 10.1074/jbc.M807354200 19097999

[B30] Ferron L. (2016). Fragile X mental retardation protein controls ion channel expression and activity. J. Physiol. 594, 5861–5867. 10.1113/JP270675 26864773 PMC5063927

[B31] Greenblatt E. J. Spradling A. C. (2018). Fragile X mental retardation 1 gene enhances the translation of large autism-related proteins. Science 361, 709–712. 10.1126/science.aas9963 30115809 PMC6905618

[B32] Gross C. Yao X. Pong D. L. Jeromin A. Bassell G. J. (2011). Fragile X mental retardation protein regulates protein expression and mRNA translation of the potassium channel Kv4.2. J. Neurosci. 31, 5693–5698. 10.1523/JNEUROSCI.6661-10.2011 21490210 PMC3089949

[B33] Guo J. U. Bartel D. P. (2016). RNA G-quadruplexes are globally unfolded in eukaryotic cells and depleted in bacteria. Science 353:6306. 10.1126/science.aaf5371 27708011 PMC5367264

[B34] Harris S. W. Hessl D. Goodlin-Jones B. Ferranti J. Bacalman S. Barbato I.. (2008). Autism profiles of males with fragile X syndrome. Am. J. Ment. Retard. 113, 427–438. 10.1352/2008.113:427-438 19127654 PMC2629645

[B35] Herman A. B. Vrakas C. N. Ray M. Kelemen S. E. Sweredoski M. J. Moradian A.. (2018). FXR1 is an IL-19-responsive RNA-binding protein that destabilizes pro-inflammatory transcripts in vascular smooth muscle cells. Cell Rep., 24, 1176–1189. 10.1016/j.celrep.2018.07.002 30067974 PMC11004729

[B36] Hernandez R. N. Feinberg R. L. Vaurio R. Passanante N. M. Thompson R. E. Kaufmann W. E. (2009). Autism spectrum disorder in fragile X syndrome: a longitudinal evaluation. Am. J. Med. Genet. A 149 A, 1125–1137. 10.1002/ajmg.a.32848 PMC2734278 19441123

[B37] Huber K. M. Gallagher S. M. Warren S. T. Bear M. F. (2002). Altered synaptic plasticity in a mouse model of fragile X mental retardation. Proc. Natl. Acad. Sci. U.S.A. 99, 7746–7750. 12032354 10.1073/pnas.122205699 PMC124340

[B38] Khalfallah O. Jarjat M. Davidovic L. Nottet N. Cestele S. Mantegazza M.. (2017). Depletion of the Fragile X mental retardation protein in embryonic stem cells alters the kinetics of neurogenesis. Stem Cells 35, 374–385. 10.1002/stem.2505 27664080

[B39] Khandjian E. W. Huot M. E. Tremblay S. Davidovic L. Mazroui R. Bardoni B. (2004). Biochemical evidence for the association of fragile X mental retardation protein with brain polyribosomal ribonucleoparticles. Proc. Natl. Acad. Sci. U.S.A. 101, 13357–13362. 10.1073/pnas.0405398101 15329415 PMC516571

[B40] Khayachi A. Gwizdek C. Poupon G. Alcor D. Chafai M. Cassé F.. (2018). Sumoylation regulates FMRP-mediated dendritic spine elimination and maturation. Nat. Comm. 9:757. 10.1038/s41467-018-03222-y 29472612 PMC5823917

[B41] Kwan K. Y. Lam M. M. Johnson M. B. Dube U. Shim S. Rasin M. R.. (2012). Species-dependent posttranscriptional regulation of NOS1 by FMRP in the developing cerebral cortex. Cell 149, 899–911. 10.1016/j.cell.2012.02.060 22579290 PMC3351852

[B42] Liu B. Li Y. Stackpole E. E. Novak A. Gao Y. Zhao Y.. (2018). Regulatory discrimination of mRNAs by FMRP controls mouse adult neural stem cell differentiation. Proc Nat Acad Sci U.S.A. 115, E11397–E11405. 10.1073/pnas.1809588115 30373821 PMC6275535

[B43] Martin K. C. Ephrussi A. (2009). mRNA localization: gene expression in the spatial dimension. Cell 136, 719–730. 10.1016/j.cell.2009.01.044 19239891 PMC2819924

[B44] Maurin T. Lebrigand K. Castagnola S. Paquet A. Jarjat M. Popa A.. (2018a). HITS-CLIP in various brain areas reveals new targets and new modalities of RNA binding by fragile X mental retardation protein. Nucleic Acids Res. 46, 6344–6355. 10.1093/nar/gky267 29668986 PMC6158598

[B45] Maurin T. Melancia F. Jarjat M. Castro L. Costa L. Delhaye S.. (2018b). Involvement of phosphodiesterase 2A activity in the pathophysiology of fragile X syndrome. Cereb Cortex. [Epub ahead of print]. 10.1093/cercor/bhy192 30137253

[B46] Maurin T. Melko M. Abekhoukh S. Khalfallah O. Davidovic L. Jarjat M.. (2015). The FMRP/GRK4 mRNA interaction uncovers a new mode of binding of the Fragile X mental retardation protein in cerebellum. Nucleic Acids Res. 43, 8540–8550. 10.1093/nar/gkv801 26250109 PMC4787806

[B47] Maurin T. Zongaro S. Bardoni B. (2014). Fragile X Syndrome: from molecular pathology to therapy. Neurosci. Biobehav. Rev. 46(Pt 2), 242–255. 10.1016/j.neubiorev.2014.01.006 24462888

[B48] Melancia F. Trezza V. (2018). Modelling fragile X syndrome in the laboratory setting: a behavioral perspective. Behav. Brain Res. 350, 149–163. 10.1016/j.bbr.2018.04.042 29704597

[B49] Melko M. Bardoni B. (2010). The role of G-quadruplex in RNA metabolism: involvement of FMRP and FMR2P. Biochimie 92, 919–926. 10.1016/j.biochi.2010.05.018 20570707

[B50] Miyashiro K. Y. Beckel-Mitchener A. Purk T. P. Becker K. G. Barret T. Liu L.. (2003). RNA cargoes associating with FMRP reveal deficits in cellular functioning in Fmr1 null mice. Neuron 37, 417–431. 10.1016/S0896-6273(03)00034-5 12575950

[B51] Nolze A. Schneider J. Keil R. Lederer M. Hüttelmaier S. Kessel M. M.. (2013). FMRP regulates actin filament organization via the armadillo protein p0071. RNA 19, 1483–1496. 10.1261/rna.037945.112 24062571 PMC3851716

[B52] Okray Z. de Esch C. E. Van Esch H. Devriendt K. Claeys A. Yan J.. (2015). A novel fragile X syndrome mutation reveals a conserved role for the carboxy-terminus in FMRP localization and function. EMBO Mol. Med. 7:423–437. 10.15252/emmm.201404576 25693964 PMC4403044

[B53] Pichon X. Lagha M. Mueller F. Bertrand E. (2018). A growing toolbox to image gene expression in single cells: sensitive approaches for demanding challenges. Mol. Cell 71, 468–480. 10.1016/j.molcel.2018.07.022 30075145

[B54] Qin M. Schmidt K. C. Zametkin A. J. Bishu S. Horowitz L. M. Burlin T. V.. (2013). Altered cerebral protein synthesis in fragile X syndrome: studies in human subjects and knockout mice. J. Cereb. Blood Flow Metab. 33, 499–507. 10.1038/jcbfm.2012.205 23299245 PMC3618394

[B55] Ramos A. Hollingworth D. Adinolfi S. Castets M. Kelly G. Frenkiel T. A. (2006). The N-terminal domain of the Fragile X Mental retardation Protein forms a novel platform for protein-protein interaction. Structure 14, 21–31. 10.1016/j.str.2005.09.018 16407062

[B56] Richter J. D. Coller J. (2015). Pausing on polyribosomes: make way for elongation in translational control. Cell 163, 292–300. 10.1016/j.cell.2015.09.041 26451481 PMC4600128

[B57] Schaeffer C. Bardoni B. Mandel J. L. Ehresmann B. Ehresmann C. Moine H. (2001). The fragile X mental retardation protein binds specifically to its mRNA via a purine quartet motif. EMBO J. 20, 4803–4813. 10.1093/emboj/20.17.4803 11532944 PMC125594

[B58] Sossin S. W. Costa-Mattioli M. (2018). Translational control in the brain in health and disease. Cold Spring Harb Perspect Biol. 6:a032912 10.1101/cshperspect.a032912 PMC6671938 30082469

[B59] Suhl J. A. Chopra P. Anderson B. R. Bassell G. J. Warren S. T. (2014). Analysis of FMRP mRNA target datasets reveals highly associated mRNAs mediated by G-quadruplex structures formed via clustered WGGA sequences. Hum. Mol. Genet. 23, 5479–5491. 10.1093/hmg/ddu272 24876161 PMC4168832

[B60] Tabet R. Moutin E. Becker J. A. Heintz D. Fouillen L. Flatter E.. (2016). Fragile X Mental Retardation Protein (FMRP) controls diacylglycerol kinase activity in neurons. Proc. Natl. Acad. Sci. U.S.A. 113, E3619–3628. 10.1073/pnas.1522631113 27233938 PMC4932937

[B61] Tang B. Wang T. Wan H. Han L. Qin X. Zhang Y.. (2015). Fmr1 deficiency promotes age-dependent alterations in the cortical synaptic proteome. Proc. Natl. Acad. Sci. U.S.A. 112, E4697–4706. 10.1073/pnas.1502258112 26307763 PMC4553823

[B62] Tomasi G. Veronese M. Bertoldo A. Beebe Smith C. Schmidt K. C. (2018). Effects of shortened scanning intervals on calculated regional rates of cerebral protein synthesis determined with the L-[1-11C] leucine PET method. PLoS ONE 13:e0195580 10.1371/journal.pone.0195580 29659612 PMC5901930

[B63] Tuorto F. Legrand C. Cirzi C. Federico G. Liebers R. Müller M.. (2018). Queuoqine-modified tRNAs confer nutritional control of protein translation. EMBO J. 37:e99777. 10.15252/embj.201899777 30093495 PMC6138434

[B64] Vasudevan S. Steitz J. A. (2007). AU-rich-element-mediated upregulation of translation by FXR1 and argonaute 2. Cell 128, 1105–1118. 10.1016/j.cell.2007.01.038 17382880 PMC3430382

[B65] Vicario A. Colliva A. Ratti A. Davidovic L. Baj G. Gricman Ł.. (2015). Dendritic targeting of short and long 3' UTR BDNF mRNA is regulated by BDNF or NT-3 and distinct sets of RNA-binding proteins. Front. Mol. Neurosci. 8:62. 10.3389/fnmol.2015.00062 26578876 PMC4624863

[B66] Westmark C. J. Sokol D. K. Maloney B. Lahiri D. K. (2016). Novel roles of amyloid-beta precursor protein metabolites in fragile X syndrome and autism. Mol. Psychiatry 21, 1333–1341. 10.1038/mp.2016.134 27573877 PMC5580495

